# Supplementary material for: The discovery of novel noncoding RNAs in 50 bacterial genomes
Source: Nucleic Acids Res. 2024 Apr 22;52(9):5152–65. doi: 10.1093/nar/gkae248 (PMC11109978; doi:10.1093/nar/gkae248)
Supplement: gkae248_Supplemental_Files [file gkae248_supplemental_files.zip › Narunsky 50 Genomes RNA Motifs Files.zip!/Publication/54-P_aestuarii/Figures/LRC-54-7-NC_011059_1_IGR_25_2484350_2484772.sto.pdf]

LRC-54-7-NC_011059_1_IGR_25_2484350_2484772.sto.r2r

5′- R C G A G C A U Y A A U C C U U R C G U U U Y R A G C C U U C A C C A U U R G Y C R A A G Y C R G G A A A A A C C Y C C U G C A A R Y G U U A Y U U C A C U A A U A A C A A Y G U C C C Y G U
